# Supplementary material for: Perovskite-Surface-Confined Grain Growth for High-Performance Perovskite Solar Cells
Source: Nanomaterials (Basel). 2022 Sep 26;12(19):3352. doi: 10.3390/nano12193352 (PMC9565253; doi:10.3390/nano12193352)
Supplement: Supplementary file 1 [file nanomaterials-12-03352-s001.zip › nanomaterials-1937071-supplementary.pdf]

nanomaterials

MDPI

Supplementary Materials

Perovskite-Surface-Confined Grain Growth for High-Performance Perovskite Solar Cells

Sajid Sajid [1,2], Salem Alzahmi [1,2,*], Imen Ben Salem [3] and Ihab M. Obaidat [2,4,*]

1 Department of Chemical & Petroleum Engineering, United Arab Emirates University, Al Ain P.O. Box 15551, United Arab Emirates
2 National Water and Energy Center, United Arab Emirates University, Al Ain P.O. Box 15551, United Arab Emirates
3 College of Natural and Health Sciences, Zayed University, Abu Dhabi P.O. Box 144534, United Arab Emirates
4 Department of Physics, United Arab Emirates University, Al Ain P.O. Box 15551, United Arab Emirates
* Correspondence: s.alzahmi@uaeu.ac.ae (S.A.); iobaidat@uaeu.ac.ae (I.M.O.)

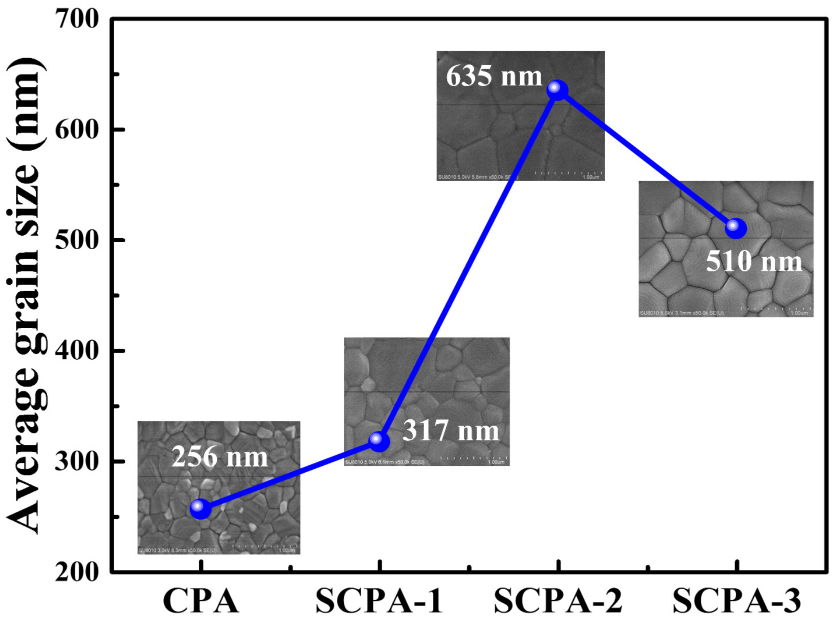

Figure S1. Estimation of average grain size of the as-prepared perovskite thin-films. Using ImageJ and a scale set to 1 μm, the average grain size was determined by dividing line length by number of grains.

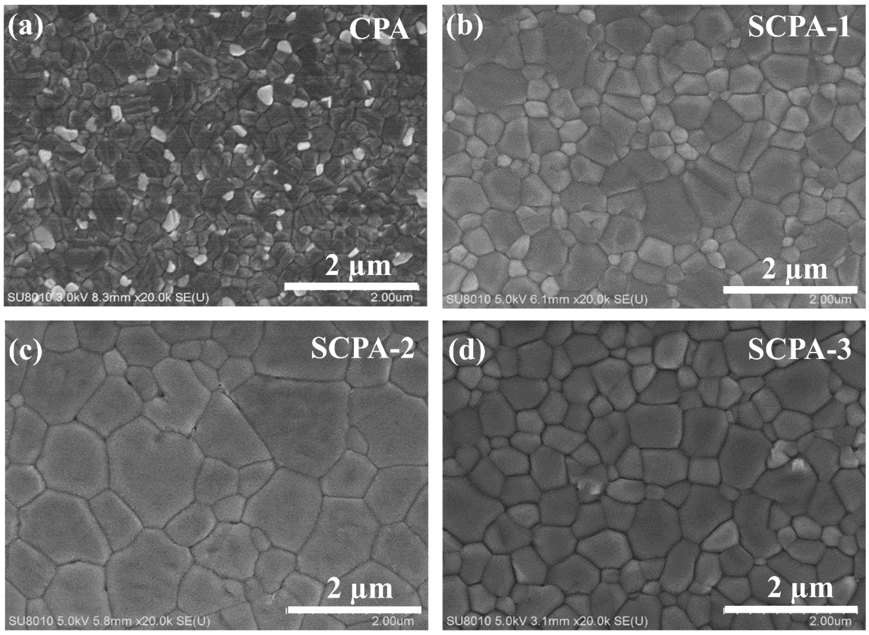

Figure S2. SEM images of the as-prepared perovskite thin-films prepared with CPA (a), SCPA (b–d).

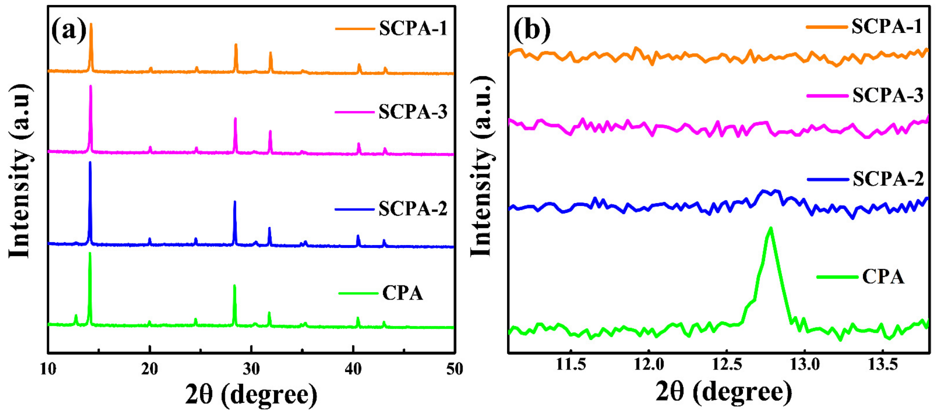

Figure S3. XRD patterns of the as-prepared perovskite thin-films prepared with CPA and SCPA (a) with zoom in at 12.7° (b).

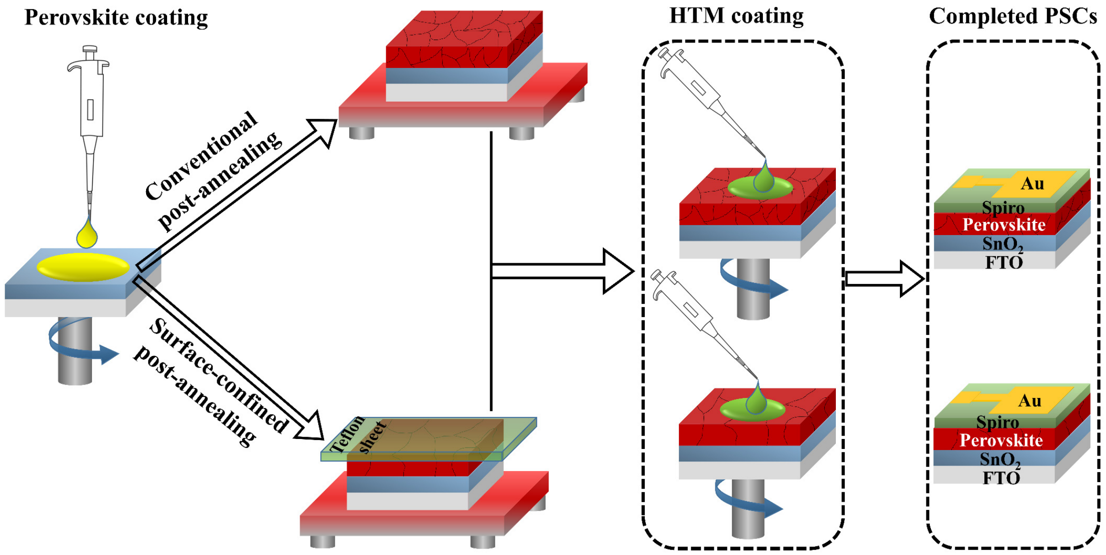

Figure S4. Schematic illustration for the preparation of PSCs with conventional post-annealing (CPA) and surface-confined post-annealing (SCPA).

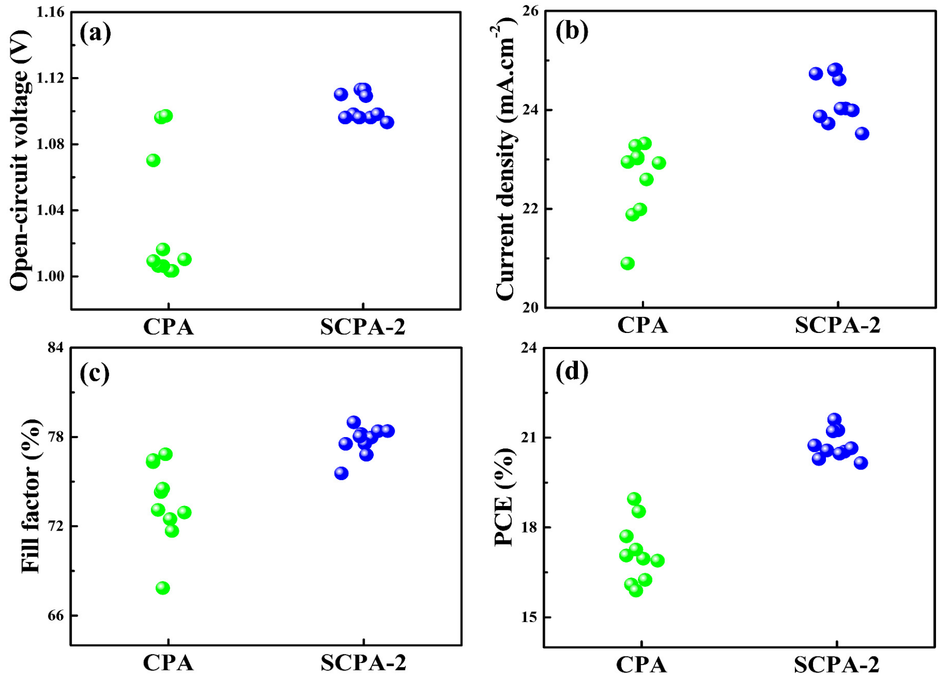

Figure S5. Statistical data of the as-prepared PSCs based on CPA and SCPA methods.

Table S1. Photovoltaic parameters (used for Figure S5) of the as-prepared PSC based on CPA and SCPA methods.

Method	V_{oc} (V)	J_{sc} (mA.cm^{-2})	FF (%)	PCE (%)
CPA	1.003	23.31356	72.4568	16.94294
---	1.003	22.58581	71.65086	16.23148
---	1.096	23.26834	74.27201	18.94092
---	1.006	23.00863	74.50609	17.24569
---	1.006	21.87205	73.07148	16.07812
---	1.097	21.97859	76.81635	18.52082
---	1.070	20.88492	76.28482	17.04726

---	1.016	23.0394	67.80729	15.87235
---	1.009	22.93871	76.4306	17.68998
---	1.010	22.91853	72.89675	16.87393
SCPA	1.113	24.81567	78.18388	21.59426
----	1.096	24.02394	77.94243	20.52243
----	1.113	24.60997	77.53495	21.23752
----	1.098	23.98265	78.37443	20.6383
----	1.098	23.71875	78.96639	20.56537
----	1.11	24.72708	75.53415	20.7319
----	1.093	23.51326	78.37803	20.14315
----	1.109	24.02252	76.7839	20.45598
----	1.096	23.86466	77.51847	20.27547
----	1.096	24.79993	78.01626	21.20538
